# Patient Survey on Satisfaction and Impact of 123I-Ioflupane Dopamine Transporter Imaging

**DOI:** 10.1371/journal.pone.0134457

**Published:** 2015-07-30

**Authors:** Matthew F. Covington, Scott Sherman, Denise Lewis, Hong Lei, Elizabeth Krupinski, Phillip H. Kuo

**Affiliations:** 1 Department of Medical Imaging, University of Arizona College of Medicine, Tucson, Arizona, United States of America; 2 Department of Neurology, University of Arizona College of Medicine, Tucson, Arizona, United States of America; 3 Department of Medical Imaging, Medicine and Biomedical Engineering, University of Arizona College of Medicine, Tucson, Arizona, United States of America; Georgia Regents University, UNITED STATES

## Abstract

Patients were surveyed to assess the impact of dopamine transporter imaging on diagnostic confidence, change in treatment plan, effect on medication compliance, and subjective well-being. Surveys were sent to 140 patients who completed dopamine transporter imaging an average of 18 months prior. Sixty-five surveys from patients (46%) were returned. Questions assessed patients’ perceived impact of the imaging on their care. Increased diagnostic confidence following imaging was reported by 69% of patients. Changes to treatment plan from imaging were reported by 24% of patients. Overall satisfaction with the study and its impact was reported by 70% of patients. Dopamine transporter imaging increased diagnostic confidence among patients and overall patient satisfaction with the impact of imaging on clinical care was high.

## Introduction

Dopamine transporter SPECT imaging using ^123^I-Ioflupane is useful and safe for differentiating adult patients with suspected Parkinson’s disease (PD) or parkinsonian syndromes (PS) from normal patients or patients with other entities including essential tremor (ET), dystonic tremor, psychogenic parkinsonism or drug-induced parkinsonism [1–7]. Diagnosis by history and clinical examination may be problematic, especially early in the disease when clinical symptoms have not fully developed or in cases with atypical features. Subsequent overdiagnosis of PD has been reported for 15–53% of cases [8–9].

^123^I-Ioflupane binds to dopamine transporters on the presynaptic terminals of dopaminergic neurons and demonstrates abnormal striatal uptake when loss of nigrostriatal dopaminergic neurons has occurred, as in cases of PD and PS such as progressive supranuclear palsy or Lewy Body dementia [7, 10]. No loss of nigrostriatal dopaminergic neurons is seen with essential tremor or normalcy [6,11]. Confirmation of PD or PS guides timely treatment whereas a normal result offers emotional relief, precludes empirical anti-parkinsonian medications, and redirects diagnostic testing in the proper direction [12–14].

Physician surveys on the impact of dopamine transporter imaging on diagnostic confidence and treatment plan changes have been performed in previous studies [15], and these factors have been studied in prospective trials [1–5,7]. This novel study is the first patient survey with long-term follow-up assessing patient satisfaction and perceived clinical impact following dopamine transporter imaging.

## Materials and Methods

This study was HIPAA compliant and deemed institutional IRB compliant by the Human Subjects Protection Program at the University of Arizona. Waiver of consent was approved by this ethics committee for this hospital quality assurance project. An 11 question patient survey assessing the perceived impact of dopamine transporter imaging on diagnostic certainty, treatment plan, and subjective well-being was created by the authors (Table 1). While the main purpose of this research was to collect data from patients, a secondary survey of referring neurologists was also performed but dependable conclusions could not be drawn from the physician survey data as 40 of the 50 responses (80%) were from a single neurologist. This data was therefore not included in this study.

**Table 1:** Patient survey questions and responses.

Questions and Responses	Percent Response	Statistical Significance
1. How long have you had your tremor or movement disorder (years and/or months)?		
*Question excluded for inability to interpret many responses.	Not applicable.	Not applicable.
2. Did the brain imaging alter your level of confidence in the diagnosis your physician provided you with?		
Much more confident	9/64 (25.7%)	Significantly more “much more confident” or “more confident” (*p* < 0.0001).
More confident	35/64 (54.7%)	
Neutral	17/64 (26.6%)	
Less confident	2/64 (0.3%)	
Much less confident	1/64 (0.2%)	
3. Did your physician change your treatment plan as a result of the brain imaging?		
Yes	15/63 (23.8%)	Significantly more “no” (*p* < 0.0001).
No	48/63 (76.2%)	
4. If yes, how was it changed?		
Another test ordered	1/18 (5.6%)	Significantly more “change in dose of medication” or “change in medication” (*p* < 0.05).
Change in medication	7/18 (38.9%)	
Change in dose of medication	10/18 (55.6%)	
5. Do you feel better because of the change?		
Yes	13/38 (34.2%)	Significantly more “no change” (*p* < 0.001).
No	3/38 (7.9%)	
No change	22/38 (57.9%)	
6. If there was a change in how you feel, over what period of time did the change occur (please be as exact as possible, for example, number of days or weeks)?		
2.7 months on average (range 2 weeks to 7 weeks; 11 responses)	Not applicable.	Not applicable.
7. Did the results of the exam affect whether you take medications for your tremor/movement disorder as recommended?		
I was not prescribed medications	13/64 (20.3%)	Significantly more “did not change” (*p* < 0.0001).
I am now more likely to take my medications as my doctorordered	15/51 (29.4%)	
I am now less likely to take my medication as my doctor ordered	2/51(3.9%)	
The results did not change the way I take my medications	34/51 (66.7%)	
8. Would you recommend the exam to other patients like yourself?		
Definitely	26/63 (41.3%)	Significantly more “definitely” or “probable” (*p* < 0.0001).
Probably	28/63 (44.4%)	
Probably Not	7/63 (11.1%)	
Definitely Not	2/63 (3.2%)	
9. How concerned are you about your exposure to radiation from the exam?		
Concerned	9/64 (14.1%)	Significantly more “unconcerned” (*p* < 0.01).
Neutral	26/64 (40.6%)	
Unconcerned	29/64 (45.3%)	
10. How did you learn of this exam?		
Primary care physician	16/63 (25.4%)	Significantly more from “other physician” (*p* < 0.0001).
Other physician	43/63 (68.3%)	
Public media	1/63 (1.6%)	
Friend/Associate	0/63 (0%)	
Other	3/63 (4.8%)	
11. What is your overall satisfaction with the experience of the study and its impact on you?		
Very satisfied	17/64 (26.6%)	Significantly more “very satisfied” or “satisfied” (*p* < 0.0001).
Satisfied	28/64 (43.8%)	
Neutral	14/64 (21.9%)	
Unsatisfied	2/64 (3.1%)	
Very unsatisfied	3/64 (4.7%)	

### Survey population

All patients with referrals from neurologists specializing in movement disorders who completed dopamine transporter imaging between July 12, 2011 and March 22, 2012 were included in this study. 140 patients were sent surveys in the mail. Patients were excluded if their referring physicians were not neurologists specializing in movement disorders. Fourteen patients were excluded.

### Data collection

Patients were instructed to return surveys through the mail in self-addressed and stamped envelopes. Each patient was surveyed only once and no patient underwent repeat dopamine transporter imaging during the study period. No financial or other form of compensation was offered to patients for completion of the survey.

Chart review was performed to assess imaging indication, imaging result and changes to pharmacologic therapy within one clinic visit following imaging.

### Imaging Technique

All imaging studies were performed at a single academic medical center. At least 1 hour prior to injection, patients were give 4 drops of Lugol’s solution in a glass of water to block radioactive iodine uptake by the thyroid gland. Imaging protocol included a 4–6 mCi (148–222 MBq) dose of ^123^I-Ioflupane administered intravenously followed by SPECT imaging 3–6 hours following injection. Each study was interpreted prospectively as part of routine clinical care by one of two board-certified radiologists with fellowship training in Nuclear Medicine.

### Statistical methods

Survey responses were entered into an electronic spreadsheet for analysis. Tabulated responses from patients and limited responses by physicians were analyzed for statistical significance by chi-squared tests, including assessment for correlation among responses between both groups. A *p*-value of less than or equal to 0.05 was considered significant.

## Results

Of 140 patient surveys, 64 patients responded corresponding to a 46% response rate. An average of 51 out of 64 patients responded to each question on the patient survey. The range of responses to each individual question ranged between 11 and 64 responses. Average patient age was 68.9 years (range 46–85 years). Males comprised 57% of the population (37/65). Patients were followed by their referring neurologist for an average of 15 months prior to imaging (range 2 weeks to 6 years; 10 responses). Patients completed dopamine transporter imaging an average of 18 months prior to completing the survey (range 14–22 months). Scans showed abnormal dopamine striatal uptake in 66.1% of cases (43/65).

The results from the first question on the patient survey that asked “How long have you had your tremor or movement disorder (years and/or months)?” could not be interpreted, as the majority of patients did not indicate years or months despite the specific wording of the question.

Of the patients responding to the survey, 68.8% (44/64) were statistically significantly either “much more confident” or “more confident” in their diagnosis following imaging compared to “neutral”, “less confident”, or “much less confident” (*p*<0.0001).

Changes in treatment plan were reported by 23.8% of patients (15/63). When asked if they felt better because of the change, 34.8% of patients answered “yes” (13/38), 7.9% answered “no” (3/38) and 57.9% answered “no change” (22 of 38). The average time period over which the improvement occurred was 2.7 months (range 2 weeks to 7 months; 11 responses). No significant difference for diagnostic confidence or treatment plan changes was found as a function of whether imaging was normal or abnormal.

For the 51 patients prescribed medications for tremor/movement disorder, 66.7% reported imaging results “did not change” medication compliance (34/51), 29.4% reported they are “more likely” to take prescribed medications as recommended (15/51) and 3.9% reported they are “less likely” to take prescribed medications as recommended (2/51). Patients were significantly more likely to take prescribed medications as recommended if their imaging test was abnormal (*p* = 0.036). As a result of imaging, a “change in medication” was reported by 38.9% of patients (7/18), a “change in dose of medication” was reported by 55.6% of patients (10/18) and “another test ordered” was reported by 5.6% of patients (1/18).

86% of patients (54/63) would “definitely” or “probably” recommend dopamine transporter imaging to similar patients. When asked regarding overall satisfaction with the experience of the study and its impact 70.3% of patients (45/64) chose “very satisfied” or “satisfied”, 21.9% of patients (14/64) felt “neutral” and 7.8% (5/64) were “unsatisfied” or “very unsatisfied”.

Patient survey results are included in their entirety in Table 1 and S1 PatientDataset. Changes to therapy by indication and result of dopamine transporter imaging are presented in Table 2.

**Table 2:** Changes in therapy by indication and result of dopamine transporter imaging.

Indication	Percentage	Imaging Result	Pharmacologic change
Clinically uncertain parkinsonism	69.2% (45/65)	32 abnormal13 normal	None 40% (18/45) Stop or reduce dopaminergic therapy 8.9% (4/45) Start dopaminergic therapy 6.7% (3/45) Increase dopaminergic therapy 6.7% (3/45) Other* 8.9% (4/45) Unknown 28.9% (13/45)
PD vs ET	21.5% (14/65)	8 abnormal6 normal	Start ET treatment 28.6% (4/14) Start or increase PD treatment 28.6% (4/14) None 21.4% (3/14) Unknown 21.4% (3/14)
PD vs MSA	6.2% (4/65)	3 abnormal1 normal	Start or increase PD treatment 50% (2/4) None 50% (2/4)
PD vs Myasthenia Gravis	3.1% (2/65)	0 abnormal2 normal	Stop PD treatment 50% (1/2) None 50% (1/2)

Abbreviations: PD = Parkinson’s disease; ET = essential tremor; MSA = multiple system atrophy; RLS = restless leg syndrome

*Start ET treatment (n = 2), adjusting antipsychotic (n = 1), starting neuromodulatory drugs (n = 1)

## Discussion

To our knowledge, this is the first patient survey with long-term follow-up obtaining responses regarding patient satisfaction and perceived impact of dopamine transporter imaging. As reported by the patients, dopamine transporter imaging significantly increased diagnostic confidence and led to changes in treatment plan for up to 57% of patients. Our results agree with existing peer-reviewed literature showing that dopamine transporter imaging leads to a change in diagnosis in 31–50% of patients [1,2,15] and changes in management for 52%-58% of patients [2,15].

At an average of 18 months following imaging more than one-third of patients with treatment plan changes in this survey reported feeling better as a result of the DAT imaging. Existing literature has failed to demonstrate related increases in total quality of life scores at either 4 weeks or 12 weeks (2) or 1-year (1) following imaging. A potential explanation for this difference is that a longer follow-up interval as in this study is needed to measure differences in quality of life.

Previous research suggests diagnostic confidence is increased only when the dopamine transporter imaging result is abnormal [3,12] and a normal result leads to decreased diagnostic confidence (3). This survey did not reveal a significant difference in diagnostic confidence among patients as a function of whether the imaging result was normal or abnormal. Patient diagnosis was significantly more likely to be changed when the imaging result was abnormal.

A recent retrospective analysis from a subspecialty movement disorder clinic in a US academic medical center reports that a change in diagnosis or medication regimen occurred in 59% of patients within one visit after dopamine transporter imaging [16]. The greatest impact on change to clinical diagnosis and/or medication regimen was found for patients with a pre-imaging diagnosis of essential tremor vs Parkinson’s disease. Our study also found that pharmacologic treatment was most likely to change for patients with the indication of essential tremor vs Parkinson’s disease.

Despite previous research demonstrating the cost-effectiveness of dopamine transporter imaging in Europe [12–14] this imaging exam currently faces serious challenges in reimbursement in the United States and, as a result, reduced availability to patients. Although this patient survey did not directly assess cost-effectiveness, findings of the survey suggest that the exam adds clinical value from the patient’s perspective. A logical next step would be to survey patients and their treating physicians to assess whether the result of dopamine transporter imaging led to future reductions in office visits, diagnostic testing, avoidance of medication expenses and/or other reduced health care expenditures. Unless data on the benefits of imaging are provided to payors, decisions based on a proper assessment of the cost to benefit ratio is not possible. Data directly from patients, the consumers of healthcare, is also crucial for calculating benefit to society. Understanding the perception of patients is therefore critical to joint efforts by patients and physicians to preserve and advance the availability of advanced imaging.

Among the limitations of this study are that a variable number of responses were received for different questions. Some questions may have been left unanswered due to suboptimal wording or difficulty with recall. While variability in responses is an inherent challenge in this type of survey research, analysis of this study could improve question formulation for future surveys. Recall bias is possible in this retrospective survey with a long follow-up period of 18 months. This long follow-up period also may have also contributed to patient dropout, thereby reducing the survey response rate.

Additionally, all referring physicians in this study were neurologists with subspecialty training in movement disorders. Previous research suggests that general neurologists are more likely to change their diagnosis following imaging compared to movement disorder specialists [2,3]. Therefore, our study may under-represent the rate of change in diagnosis that would be found in a population of patients referred from general neurologists or primary care physicians.

A complete analysis of responders compared to non-responders was not performed. However, survey respondents were divided into a 2:1 ratio of abnormal:normal scan results which is consistent with published data on overall scan results from our patient population [17]. This suggests that likelihood of response to the survey was not a function of whether the scan result was normal or abnormal.

## Supporting Information

S1 PatientDataset Raw patient survey data. (XLSX)
